# Impairment of Lamin A/C-Polycomb crosstalk as a possible epigenetic cause of Emery Dreifuss Muscular Dystrophy (EDMD)

**DOI:** 10.1186/1750-1172-10-S2-O10

**Published:** 2015-11-11

**Authors:** Chiara Lanzuolo

**Affiliations:** 1 Institute of Cell Biology and Neurobiology (IBCN)-CNR Epigenetics and Nuclear structure Laboratory at Santa Lucia Foundation, Roma, Italy

## 

Traditionally, studies on EDMD have focused on genetic changes affecting molecules involved in the development of pathology. However, emerging findings indicate that a single genetic mutation can be accompanied by a range of different phenotypes, suggesting a contribution of the epigenetic background to the disease progression. This is in line with recent works showing that changes in chromatin architecture are peculiar of several laminopathies[1,2]. Despite much effort has been done to understand the regulation of the complex networks of gene expression that govern muscle differentiation and that is affected in EDMD, little is known about the epigenetic players and molecular mechanisms involved in pathogenesis and progression. Key epigenetic regulators of chromatin architecture are Polycomb group (PcG) of proteins, epigenetic transcriptional repressors of genes primarily involved in differentiation and development[3]. In particular, during myogenesis, modulation of Ezh2 levels, the catalytic subunit of the Polycomb Repressive Complex 2 (PRC2) ensures the correct muscle differentiation[4]. In the nucleus, PcG proteins form microscopically visible foci and high-through-put data together with microscopy analysis revealed that their targets are organized in chromatin loops[5,6]. We have shown that Lamin A/C sustains PcG foci influencing PcG nuclear compartmentalization and modulating their repressive functions. During myogenesis, Lamin A/C depletion leads to an altered timing of muscle differentiation due to the aberrant expression of PcG-regulated genes.
